# First report on the prevalence of *Fasciola hepatica* in the endangered Père David’s deer (*Elaphurus davidianus*) in China

**DOI:** 10.1186/s12917-020-02694-9

**Published:** 2020-12-03

**Authors:** Si-Yang Huang, Jing-Zhi Gong, Yi-Jun Ren, Ming Pan, Wei-Min Cai, Yi-Min Fan, Na Yao

**Affiliations:** 1 Institute of Comparative Medicine, College of Veterinary Medicine, Yangzhou University, and Jiangsu Co-innovation Center for Prevention and Control of Important Animal Infectious Diseases and Zoonosis, and Jiangsu Key Laboratory of Zoonosis, Yangzhou, Jiangsu Province 225009 People’s Republic of China; 2 Joint International Research Laboratory of Agriculture and Agri-Product Safety, the Ministry of Education of China , Yangzhou UniversityYangzhou University, Jiangsu Province Yancheng, People’s Republic of China; 3 Dafeng Elk National Natural Reserve, Yangzhou, Jiangsu Province People’s Republic of China

**Keywords:** *Fasciola hepatica*, *Elaphurus davidianus*, Nest-PCR, Prevalence, Internal transcribed spacer 2 (ITS-2)

## Abstract

**Background:**

*Fasciola hepatica* is an important zoonotic parasite that causes fasciolosis in a broad range of animals. No information is available about the prevalence of *F. hepatica* in Père David’s deer (*Elaphurus davidianus*), an endangered species in the world. Therefore, the purpose of the study was to evaluate the prevalence of fasciolosis in Père David’s deer in the Dafeng Elk National Natural Reserve, Jiangsu province, China.

**Results:**

In this study, 142 fecal samples from Père David’s deer were analyzed for *F. hepatica* by microscopy and nest-PCR. Only one sample was positive for *F. hepatica* according to microscopy examination, while 18 of 142 (12.68, 95%CI: 2.841–22.45%) samples were positive for *F. hepatica* according to nest-PCR results.

**Conclusions:**

This is the first report of prevalence of *F. hepatica* in Père David’s deer. The prevalence data indicated that *F. hepatica* was also present in this endangered animal, which may cause a potential threat to this precious species.

## Background

Fasciolosis is an important zoonotic disease, which can infect human and various animals, widely distributed in different countries in the world. It is estimated that this disease costs €2.5 billion economic losses in the global livestock production industry every year [1]. What’s worse, overuse of triclabendazole (TCBZ) results in drug resistance in *Fasciola* spp. The anti-TCBZ strains had emerged in the Netherlands, Chile, Turkey and Peru, which had a huge impact on disease prevention and control [2]. In China, the prevalence of fasciolosis in animals was quite high, 87.35% of buffaloes were infected by *F. gigantic* in Guangxi province, and 28.7% of Yaks were positive for *F. hepatica* in Gansu province [3, 4]. However, no information is available about *F. hepatica* infections in Père David’s deer.

Père David’s deer is an endangered deer species in the world, mainly distributed in China, the United States and the United Kingdom now. In 1986, 42 Père David’s deer (13 males, 29 females) were re-introduced to the Dafeng Elk National Natural Reserve from UK, and the number of Père David’s deer has been steadily increasing, reaching to 4556 in 2018. Here, we reported the prevalence of *F. hepatica* in Père David’s deer in the Dafeng Elk National Natural Reserve. To our knowledge, this is the first prevalence of *F. hepatica* infection in these endangered animals.

## Results

### Faecal examination

**Fig. 1:**
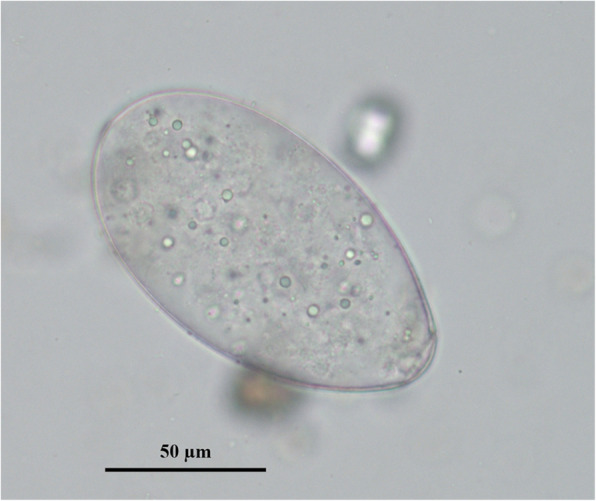
Fecal microscopy examination. The No. 86 feces sample was treated by standard sedimentation method Microscopic observation multiple was × 400. The width of the egg is 68.885 μm and the length is 124.51 μm. Scale bars: 50 μm

According to microscopy investigation result, *F. hepatica* eggs were only detected in one sample, with a size of about 68 × 124 μm (Fig. 1). Therefore, the prevalence of infection was only 0.70% (1/142), much lower than that in other species.

### Qualitative evaluation of nest-PCR tests

**Fig. 2:**
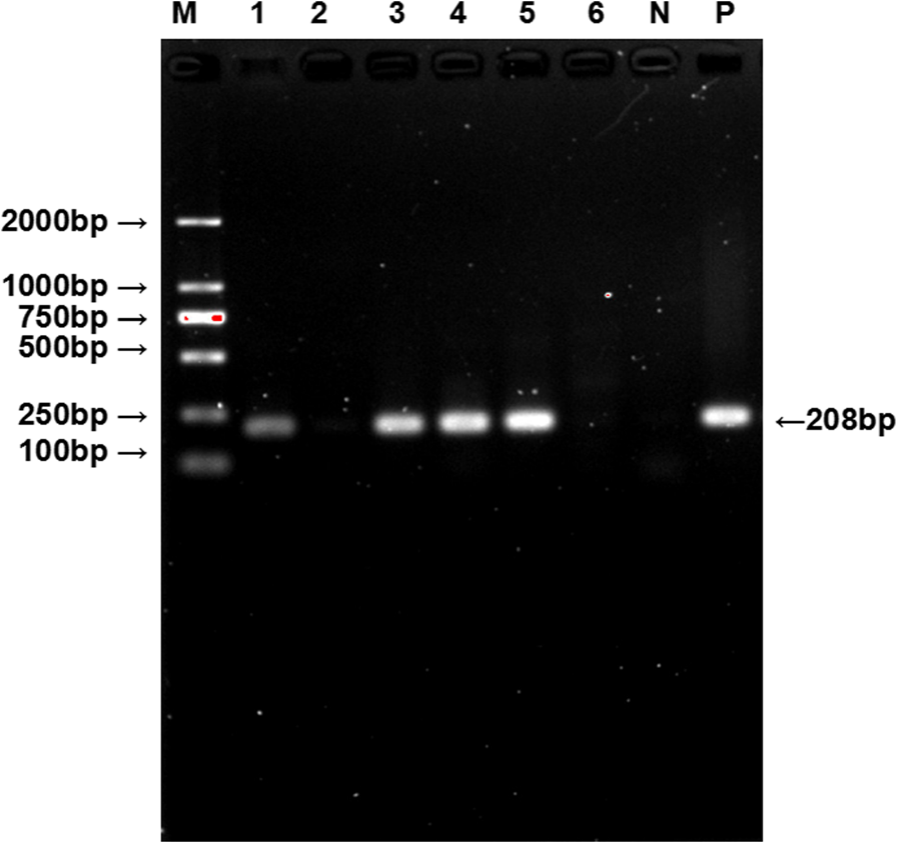
Fecal samples were detected by the nest-PCR and PCR gel electrophoresis results of some samples. Lane 1–6: The result of second PCR run No. 17, 30, 45, 64, 86 and 98 DNA sample; N: PBS; P: positive DNA. M: 2000 bp DNA Marker (TaKaRa, Japan). The result showed No.17, 45, 64 and 86 samples were positive with a distinctly single band about 208 bp, No.30 and 98 samples were negative without any band

One hundred forty-two Père David’s deer fecal genomic DNA samples were examined by the nest-PCR with *F. hepatica* genomic DNA and PBS buffer used as positive and negative controls, respectively. There was a clear band at 200 bp in the positive samples detected by nest-PCR (Fig. 2). The PCR product of positive samples were sequenced, and then confirmed by blasting in Genebank. All sequences showed 100% identity to the standard *F. hepatica* 5.8S ribosomal RNA gene sequence (GenBank: MH715295.1), confirming that these samples are all infected by *F. hepatica*. To conclude, among 137 samples, 18 were positive for *F. hepatica*, and the prevalence is as high as 12.67%(95%CI:2.841–22.45%). Among the 18 positive samples, 6 samples were collected in 2017 and 12 samples were collected in 2018, the prevalence was 13.64% (95%CI:3.496–23.77%) and 12.24% (95%CI:2.556–21.92%), respectively (Table 1). There was no significant difference in the prevalence of these 2 years (*P* > 0.05).

**Table 1:** Prevalence of *F. hepatica* infection of Dafeng Elk National Natural Reserve, in China

Year	No.Positive	No.Negative	Prevalence(%) (95% CI)	*P* value
2017	6	38	13.64%(3.496–23.77%)	> 0.05^a^
2018	12	86	12.24%(2.556–21.92%)	
Total	18	124	12.67%(2.841–22.45%)	

^a^The result was calculated by χ2 test using SPSS21 (SAS Institute Inc., Cary, North Carolina, USA). All tests were two sided

## Discussion

The Dafeng Elk National Natural Reserve is in Jiangsu province, with a warm and pleasant climate and abundant water source, and the environment provides favorable conditions for survival and reproduction for the intermediate host of *F. hepatica*. Fasciolosis is an important disease for ruminants, so the prevalence of *F. hepatica* infection was evaluated in Chinese Père David’s deer. In our study, only one sample was positive for the *F. hepatic* examined by fecal examination, while 18 samples were positive according to the nest-PCR analysis. The results showed that nest-PCR is more sensitive than the fecal examination. Therefore, in order to get better results, more sensitive detection methods like nest-PCRs should be chosen for fasciolosis surveillance in future.

Previous studies found that the prevalence of *F. hepatica* was 4.6% in Australia sika deer (*Cervus nippon*) [5], 29% in Spain roe deer (*Capreolus capreolus*) [6], 14.8% in Argentinared deer (*Cervus elaphus*) [7], and 70% in Ireland Fallow deer (*Dama dama*) [8], respectively. Our results showed that the prevalence is 12.67% in Père David’s deer in the Dafeng Nature Reserve, which was lower than Spain roe deer, Argentinared deer and Ireland Fallow deer, but higher than Australia sika deer. The prevalence differences might due to the detection methods, living environment and other different factors. Although the Père David’s deer in this study were wild animals, comparing to other wild animals, they were raised in closed area, having less chance of infection, because other ruminants were not allowed to enter this area. Given the reasons above, contaminated water or infected snails might play specific roles in the prevalence of *F. hepatica* infection.

Although there is no case about the anemia, diarrhea or death of Père David’s deer caused by *F. hepatica* infection. Fasciolosis was reported in other deer species and bile duct lesions that has been linked to *F. hepatica* in wild sika deer in Japan [9]. Because Père David’s deer like swimming, the Dafeng Nature Reserve located in swampland, the environment is suitable for snail reproduction and *F. hepatica* eggs development. This is a warning for the prevalence of fasciolosis in Père David’s deer. It is important to carry out surveillance about fasciolosis in the Dafeng Elk National Natural Reserve. This study for the first time conducted molecular surveillance of the *F. hepatica* in Père David’s deer, and provided basic data for this disease in this area.

## Conclusions

In conclusion, this is the first prevalence of *F. hepatica* infection in wild Père David’s deer. These results will provide useful information for establishing surveillance programs and basic data for future research on *F. hepatica* infection of Père David’s deer.

## Methods

### Ethics statement

This study was approved by the Animal Ethics Committee of Yangzhou University and the sample collection was permitted by Ethics Committee of Dafeng Elk National Natural Reserve. All Père David’s deer samples were handled in accordance with good animal practices required by the Animal Ethics Procedures and Guidelines of the People’s Republic of China.

### Study area and sample collection

The present study was conducted in the Dafeng Elk National Natural Reserve (120.46′44.66″ ~ 120.53′26.60″E, 32.58′31.67″ ~ 33.03′27.60″N), which is located on the shore of the Yellow Sea. There are the largest wild elk population and largest elk gene bank in the world [10], and this area is very good for the elk living.

A total of 142 stool samples were randomly collected from August 2017 to August 2018. The fresh samples were kept in a cold box and immediately transported to the Laboratory. The fecal samples were divided into two, the first part was stored at 4 °C until egg counting techniques were performed within 3 days and the other part was stored at − 20 °C for extracting the genomic DNA.

### Faecal examination

The presence of *Fasciola spp.* eggs in faecal samples was evaluated by a sedimentation-flotation technique according to previous research [11] . Briefly, approximately 10 g of feces were mixed with 200 ml water and filtered 3 times to get rid of large particles. The filtrate was centrifuged at 700×*g* for 4 min, and the supernatant was discarded, the sediment was resuspended in zinc chloride and centrifuged at 180×*g* for 3 min. The floating material was collected underneath a cover slip that could stand on the test tube for 2 min. Finally, the slides were microscopically investigated under a 400 × magnification.

### Genomic DNA extraction and nest-PCR test

200 mg purified stool sample was included in each reaction, then the genomic DNA was extracted according to the Stool DNA kit (Omega D4015–02) instructions. DNA concentration and quality were measured by a Nanodrop 2000 spectrophotometer (Thermo, USA). The DNA samples were stored at − 20 °C for further research or immediately used for nest-PCR. 12.5 μL 2× Premix Taq™ (TaKaRa, China), 1 μL template DNA, 2 μL primers and 9.5 μL ddH_2_O were included in each 25 μL reaction. The reaction conditions were performed according to previous study [12]. Briefly, the cycling conditions were started with a denaturation at 95 °C 5 min, followed by 35 cycles of 30 s at 95 °C, 30 s at 56 °C (first run) or 57 °C (second run) and 40 s at 72 °C, stopped by a final extension at 72 °C for 7 min. For the first run, the sample genomic DNA was used for template, Fh-F- ATATTGCGGCCATGGGTTAG and Fh-R- CCAATGACAAAGTGACAGCG were used for primers, and for second run, template was changed into the PCR product of the first run, and the primers was replaced by n-F-TATCACGACGCCCAAAAAGTC and n-R- GATCGCCAAACACACTGACA. The genomic DNA of *F. hepatica* (160 ng/μL) and PBS buffer were used as positive control and the negative control, respectively. Amplification products were observed under UV light after electrophoresis in 3% agarose gel containing GoldView™ (Solarbio, China), and confirmed by DNA sequencing.

### Statistical analysis

The variation in *F. hepatica* prevalence of Père David’s deer from different years was calculated by χ2 test using SPSS21 (SAS Institute Inc., Cary, North Carolina, USA). All tests were two sided, and value of *P* < 0.05 was considered statistically significant, otherwise the correlation of infection rate in 2 years is not significant [4].

## Data Availability

The datasets used and analyzed during the current study are available from the corresponding author on reasonable request.

## Abbreviations

TCBZ: Triclabendazole
ITS-2: Internal transcribed spacer 2
PBS: Phosphate buffered saline
